# Efficacy and Safety Profile of Histone Deacetylase Inhibitors for Metastatic Breast Cancer: A Meta-Analysis

**DOI:** 10.3389/fonc.2022.901152

**Published:** 2022-05-31

**Authors:** Changjun Wang, Yan Lin, Hanjiang Zhu, Yidong Zhou, Feng Mao, Xin Huang, Qiang Sun, Chenggang Li

**Affiliations:** ^1^ Department of Breast Surgery, Peking Union Medical College Hospital, Beijing, China; ^2^ Department of Dermatology, University of California San Francisco, San Francisco, CA, United States; ^3^ State Key Laboratory of Medicinal Chemical Biology, Nankai University, Tianjin, China; ^4^ College of Pharmacy, Nankai University, Tianjin, China

**Keywords:** histone deacetylase inhibitors, metastatic breast cancer, entinostat, tucidinostat, endocrine therapy

## Abstract

**Introduction:**

Acquired resistance to endocrine therapy (ET) remains a big challenge in the management of metastatic breast cancer (MBC). A novel therapeutic agent, histone deacetylase inhibitors (HDACi), targets the abnormal epigenetic modification and may overcome acquired resistance. However, HDACi efficacy and the safety profile for hormone receptor (HoR)-positive/human epidermal growth factor receptor 2 (HER2)-negative MBC remain controversial.

**Methods:**

Two independent reviewers searched PubMed, Embase, and Cochrane Central Register of Controlled Trials databases for relevant studies on HDACi and HoR+/HER2- MBC. Demographic and clinicopathological parameters were extracted and presented as means and proportions, and between-group differences were assessed by Pearson chi-square test. Fixed- or random-effects models were used for meta-analysis based on inter-study heterogeneity. Pooled results were presented as L’Abbé plot and forest plot. Funnel plot and Egger’s test were employed for evaluation of publication bias.

**Results:**

Four studies with 1,457 patients were included for meta-analysis. The overall objective response rates (ORRs) of HDACi + ET (HE) and placebo + ET (PE) groups were 11.52% and 6.67%, respectively. The HE regimen significantly increased ORR (odds ratio [OR] 1.633, 95% confidence interval [CI] = 1.103–2.418, *p* < 0.05) and showed higher clinical benefit rate (CBR) than the PE regimen (HE vs. PE groups: 38.82% vs. 30.58%, OR 1.378, 95% CI = 1.020–1.861, *p* < 0.05). Additionally, the HE regimen was associated with prolonged progression-free survival (PFS) (hazard ratio [HR] 0.761, 95% CI = 0.650–0.872, *p* < 0.001) and overall survival (OS) (HR 0.849, 95% CI = 0.702–0.996, *p* < 0.001). Regarding safety profile, the HE regimen had increasing toxicity in terms of higher overall adverse event (AE), Grade ≥3 AE, dose modification, and discontinuation rate.

**Conclusions:**

This meta-analysis validated that the HE regimen had superior efficacy over control in terms of ORR, CBR, PFS, and OS, but was accompanied with increasing toxicity. HDACi plus ET could serve as an important option in managing HoR+/HER2- MBC. Future studies may focus on the clinical difference among different HDACi and AE managements to enhance tolerability.

## Introduction

Endocrine therapy (ET) is the keystone in the management of hormone receptor (HoR)-positive/human epidermal growth factor receptor 2 (HER2)-negative metastatic breast cancer (MBC). However, acquired resistance to ET remains a significant challenge; a large proportion of patients inevitably develop recurrence and lead to treatment escalation. Recently, the emergence of novel agents targeting resistance mechanism sheds light on HoR+/HER2- MBC management. The combination strategies of ET and other target therapies, such as everolimus + exemestane, aromatase inhibitor (AI)/fulvestrant + cyclin-dependent kinase 4/6 inhibitor (CDKi), and PI3K inhibitor + ET, achieved great success for HoR+/HER2- MBC treatment (1–4), even though drug resistance ultimately develops and urges further broadening of treatment portfolio.

The underlying mechanisms of acquired resistance to ET include loss of function and mutated estrogen receptor, upregulation of other growth factor-related signal pathways, cyclin D1 overexpression, and DNA methylation (5–8). Epigenetic dysregulation in cancer includes DNA methylation and histone modifications, which both lead to chromatin remodeling (9). Histone deacetylases, histone methyl transferases, and DNA methyl transferases are the main enzymes that regulate the chromatin conformation (10). Based on the mechanism that epigenetic modification could confer ET resistance, histone deacetylase inhibitors (HDACi) including valproic acid, entinostat, vorinostat, and tucidinostat were invented. The key processes regulated by HDACi include cell-cycle arrest, chemo-sensitization, apoptosis induction, and upregulation of tumor suppressors (11, 12). HDACi exhibited an antineoplastic effect *via* multiple mechanisms, such as restoring p53 transcription (13) and inducing apoptosis (14). Moreover, it also had potent anti-angiogenic and anti-metastatic activities (15, 16), as well as involvement in the reactive oxygen species metabolism, and accelerated the eradication of cancer cells (17).

However, the efficacy of HDACi remains controversial. A Phase II study by Munster et al. suggested that the combination of vorinostat and tamoxifen was well tolerated and exhibited encouraging activity in reversing hormone resistance with 19% objective response rate (ORR) and 40% clinical benefit rate (CBR) (18). Similarly, a study by Yardley et al. and a study by Jiang et al. proved that two HDACi, entinostat and tucidinostat, could both prolong progression-free survival (PFS) with acceptable tolerability (19, 20). In contrast, a study by Connolly et al. showed no improvement of survival in AI-resistant advanced HoR+/HER2- breast cancer with the combination of entinostat and exemestane (21).

Thus, the present meta-analysis included four studies with 1,457 patients to evaluate the efficacy and safety profile of HDACi on HoR+/HER2- MBC.

## Methods

### Literature Search

Literature search was performed in PubMed (from 1946 to February 2022), Embase (from 1947 to February 2022, hosted by Ovid), and Cochrane Central Register of Controlled Trials (CENTRAL, from 2000 to February 2022) databases. The following medical subject headings and keywords were used for literature search: “Histone deacetylases inhibitor”, “HDAC inhibitor”, “Vorinostat”, “Tucidinostat”, “Chidamide”, “Entinostat”, “Metastatic breast cancer”, and “Advanced breast cancer”. No limitation was set regarding languages or regions of publications. All the references were retrieved to ensure the sensitivity of the literature search and manually screened to select relevant studies.

### Selection Criteria and Quality Assessment

To be eligible, studies should meet the following inclusion criteria: studies on metastatic HoR+/HER2- breast cancer; studies on HDACi combined with ET; comparison between HDACi + ET (HE) and placebo + ET (PE); and available data for efficacy and adverse effect (AE) analyses. Exclusion criteria were set as follows: studies in neoadjuvant/adjuvant setting; single-arm studies; studies on the other breast cancer subtypes, such as triple-negative breast cancer or HER2-rich subtype; studies on HDACi combined with treatments other than ET; and review, meta-analysis, editorial, letter, case reports, guidelines, and study protocols. Two independent reviewers (CW and YL) assessed the eligibility of studies according to the above inclusion/exclusion criteria. The initial evaluation was through manual screening of titles and abstracts of all the references. Then, for potentially relevant studies, the full text of publications were retrieved and carefully reviewed by the same two reviewers. Disagreement was resolved by consensus (CW, YL, CL, and QS).

Quality assessment of the included studies was performed according to the STROBE checklist (22, 23). An ordinal scale from 1 to 5 (1 = worst, 5 = best) was used to score each item in the STROBE Checklist by two independent reviewers (CW and YL). The final quality scores were the mean of scores generated by each reviewer with higher values indicating a better methodological quality (24).

### Data Extraction

A predesigned data extraction form was used by two reviewers (CW and YL) for data collection. The characteristics of included studies (authors, publication year, country, clinical trial phases, study population, menopausal status, prior ET/chemotherapy, HDACi, number of patients included, and median follow-up), clinicopathological parameters of study population, efficacy data (ORR, CBR, PFS, and overall survival [OS]), and AE data (all AE, Grade ≥3 AE, dose modification [DM] due to AE, and discontinuation due to AE) were extracted for meta-analyses. If the data of interest were not reported in the manuscripts or abstracts, the corresponding author and first author were contacted for detailed information. Survival data (hazard ratio [HR] and 95% confidence interval [CI]) were either extracted directly from tables/figures/text of included studies or estimated from Kaplan–Meier curves using the method provided by Tierney et al. (25).

### Statistical Analysis

The demographic and clinicopathological parameters were presented as means and proportions. Between-group differences were assessed by Pearson chi-square test. Heterogeneity was presented by Cochrane’s *Q* and *I* ^2^ statistics. For *I* ^2^ statistics, *I* ^2^ < 25% was considered as low heterogeneity and *I* ^2^ > 75% was considered as high heterogeneity. Data were analyzed with a fixed-effects model for Cochrane’s *Q* test with *p* > 0.05; otherwise, the random-effects model was applied. For binary outcomes, the L’Abbé plot was used to visually display meta-analysis results of comparison between treatment and control intervention. In the L’Abbé plot, the summary outcome measures were plotted as circles with their sizes proportional to study precisions, and it also contained a reference (diagonal) line indicating identical outcomes in the two groups. Funnel plot symmetry and Egger’s test were used to assess publication bias. For endpoints with significant publication bias, “trim-and-fill” analysis was adopted to estimate the number of studies potentially missing from a meta-analysis due to publication bias and its impact on overall effect-size.

All the statistical tests were two-sided, and statistical significance was defined as *p* < 0.05. Statistical analyses were conducted by STATA version 16.0 (Stata Corporation, College Station, TX, USA).

## Results

**Figure 1:**
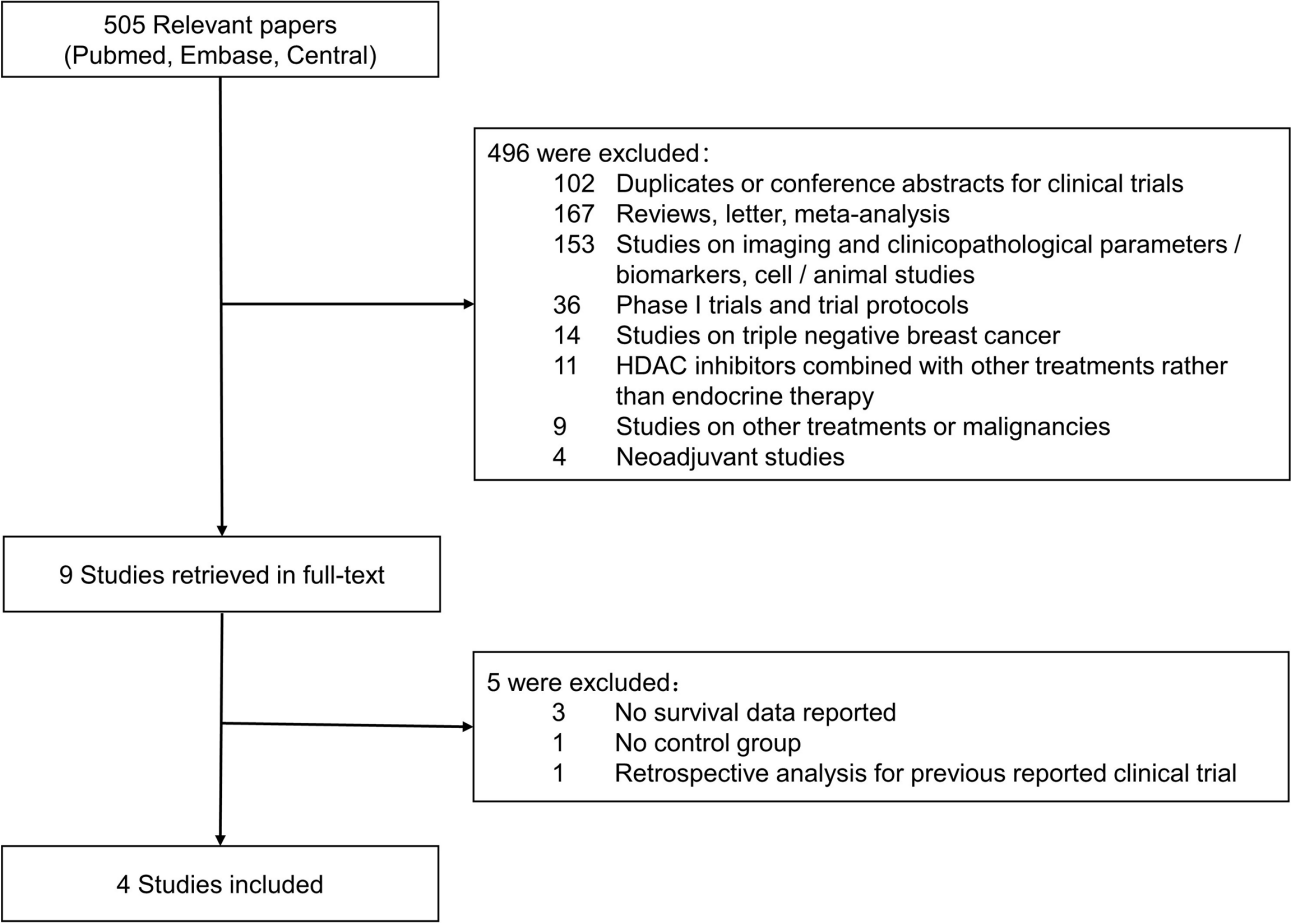
Flowchart of articles reviewed and included in the meta-analysis.

Five hundred and five relevant citations were extracted from PubMed, Embase, and CENTRAL Database, and 496 citations were excluded after initial screening according to inclusion/exclusion criteria. Nine publications were considered to be potentially relevant to the study objective and full-text articles were retrieved for further evaluation. Finally, four studies with a total of 1,457 patients were included for meta-analyses (19–21, 26). The result of literature search and screening was presented as a flowchart in **Figure 1**. **Supplementary Table 1** showed quality scores of included studies.

### Characteristics of Included Studies and Study Population

The main characteristics of included studies are summarized in **Table 1**. The four studies were three Phase III clinical trials and one Phase II trial (19). Two trials recruited exclusively post-menopausal women (19, 20), while the others enrolled both pre- and post-menopausal patients (21, 26). Only the study by Jiang et al. used tucidinostat as HDACi while all the other trials focused on entinostat (20). Patient randomization had a 2:1 ratio in the studies by Jiang et al. and Xu et al. (20, 26), while for the other trials, it was 1:1. The demographic and clinicopathological characteristics of the study population are listed in **Table 2**. All the parameters including ECOG score, visceral diseases, sensitivity to prior ET, prior CDKi, prior chemotherapy, and fulvestrant were comparable between HE and PE groups.

**Table 1:** Characteristics of studies included in the meta-analysis.

Study	Country	Phase	Study population	Menopausal status	Prior ET	Prior CT lines	HDACi	No. (HE/PE)	FU
**Yardley 2013** (19) **(ENCORE 301)**	Czech Republic, United States, Russia	**II**	Progression on NSAI (Adjuvant ET >12 m; metastatic ET > 3 m)	Post-	NSAI	≤1	Entinostat	130 (64/66)	19.5 m vs. 17.2 m
**Jiang 2019** (20) **(ACE)**	China	**III**	Progression on ≥ 1 line ET (adjuvant/metastatic setting)	Post-	Anti-Estrogen, AI, Fulvestrant	≤1	Tucidinostat	365 (244/121)	13.9 m
**Connolly 2021** (21) **(E2112)**	United States, South Africa	**III**	Progression on NSAI in adjuvant (progression on or within 12 m of completion) or metastatic setting	Pre-/post-	NSAI, Fulvestrant, Everolimus, CDKi	≤1	Entinostat	608 (305/303)	NR
**Xu 2022** (26)	China	**III**	Progression on previous ET	Pre-/post-	NS, Fulvestrant, CDKi	NS	Entinostat	354 (235/119)	NR

AI, aromatase inhibitor; CDKi, cyclin-dependent kinase 4/6 inhibitor; CT, chemotherapy; ET, endocrine therapy; FU, follow up; HDACi, histone deacetylase inhibitor; HE, HDACi + ET; NR, not reported; NSAI, non-steroid aromatase inhibitor; NS, not specified; PE, placebo + ET.

**Table 2:** Demographic and clinicopathological characteristics of the study population.

		HDACi + ET(*N* = 848)	Placebo + ET(*N* = 609)	*p*-value
**ECOG performance score**				
	**0**	439 (51.8%)	321 (52.7%)	0.763
	1	409 (48.2%)	288 (47.3%)	
**Visceral diseases**				
	Yes	521 (61.4%)	367 (60.3%)	0.690
	No	327 (38.6%)	242 (39.7%)	
**Sensitive to previous ET**				0.963
	Yes	366 (67.4%)	205 (67.0%)	
	No	177 (32.6%)	101 (33.0%)	
**Prior chemotherapy**				0.846
	Yes	554 (65.3%)	394 (64.7%)	
	No	294 (34.7%)	215 (35.3%)	
**Prior CDKi**				0.494
	**Yes**	128 (23.7%)	109 (25.8%)	
	No	412 (76.3%)	313 (74.2%)	
**Prior fulvestrant**				0.380
	**Yes**	169 (21.6%)	129 (23.8%)	
	**No**	615(78.4%)	414 (76.2%)	

HDACi, histone deacetylase inhibitor; ECOG, Eastern Cooperative Oncology Group; ET, endocrine therapy; CDKi, cyclin-dependent kinase 4/6 inhibitor.

### Pooled Results for Efficacy Endpoints of HDACi + ET in HoR+/HER2- MBC

**Figure 2:**
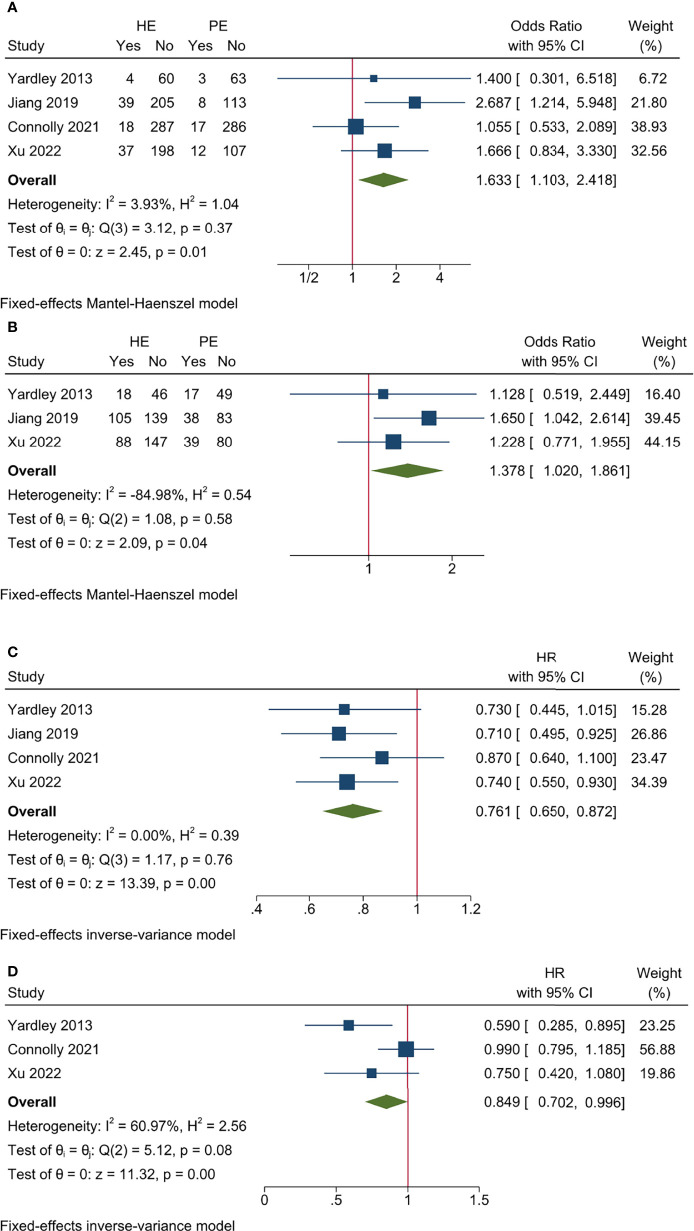
Pooled results for efficacy endpoints of included studies. **(A)** ORR; **(B)** CBR; **(C)** PFS; **(D)** OS.

All the studies reported ORR data and no significant heterogeneity existed among included studies (*I* ^2^ = 3.93%, Cochrane’s *Q p* = 0.37). The overall ORR was 11.52% and 6.67% for HE and PE groups, respectively, and the HE regimen significantly increased ORR (odds ratio [OR] 1.633, 95% CI = 1.103–2.418, *p* < 0.05) (**Table 3** and **Figure 2A**). The L’Abbé plot is presented in **Figure 4A**.

**Table 3:** Survival and adverse effect data of included studies.

Study	ORR		CBR		PFS (HE vs. PE)	OS (HE vs. PE)	All AE		Grade ≥3 AE		Dose modification		Discontinuation	
	HE	PE	HE	PE			HE	PE	HE	PE	HE	PE	HE	PE
**Yardley 2013** (19) **(ENCORE 301)**	6.30%	4.60%	28.10%	25.80%	4.3 vs. 2.3m	28.1 vs. 19.8m	95%	85%	50%	26%	35%	6%	11%	2%
**Jiang 2019** (20) **(ACE)**	16%	7%	43%	31%	9.2 vs. 3.8m	Not mature	99%	89%	75%	16%	33%	2%	8%	0%
**Connolly 2021** (21) **(E2112)**	5.80%	5.60%	NA	NA	3.3 vs. 3.1m	23.4 vs. 21.7m	NA	NA	51%	16%	30%	3%	16%	8%
**Xu 2022** (26)	15.70%	10.10%	37.40%	32.80%	6.3 vs. 3.7m	NA	99.10%	88.20%	65.60%	19.30%	NA	NA	NA	NA
**Overall**	11.52%	6.65%	38.82%	30.58%			98.57%	87.83%	61.88%	17.73%	31.72%	3.16%	12.29%	5.22%

AE, adverse effect; CBR, clinical benefit rate; HE, histone deacetylase inhibitor + endocrine therapy; NA, not available; ORR, objective response rate; PE, placebo + endocrine therapy.

Three studies had CBR data and no significant heterogeneity existed among included studies (*I* ^2^ = −84.98%, Cochrane’s Q *p* = 0.58). The overall CBR was 38.82% and 30.58% for HE and PE groups, respectively, and the HE regimen significantly increased CBR (OR 1.378, 95% CI = 1.020–1.861, *p* < 0.05) (**Table 3** and **Figure 2B**). The L’Abbé plot is presented in **Figure 4B**.

All the studies reported PFS data and no significant heterogeneity existed among included studies (*I* ^2^ = 0.00%, Cochrane’s *Q p* = 0.76). The HE regimen was associated with prolonged PFS (hazard ratio [HR] 0.761, 95% CI = 0.650–0.872, *p* < 0.001) (**Table 3** and **Figure 2C**).

Three studies had OS data and no significant heterogeneity existed among included studies (*I* ^2^ = 60.97%, Cochrane’s *Q p* = 0.08). The HE regimen had a marginal effect that could lower overall mortality (HR 0.849, 95% CI = 0.702–0.996, *p* < 0.001) (**Table 3** and **Figure 2D**).

### Pooled Results for Safety Endpoints of HDACi + ET in HoR+/HER2- MBC

**Figure 3:**
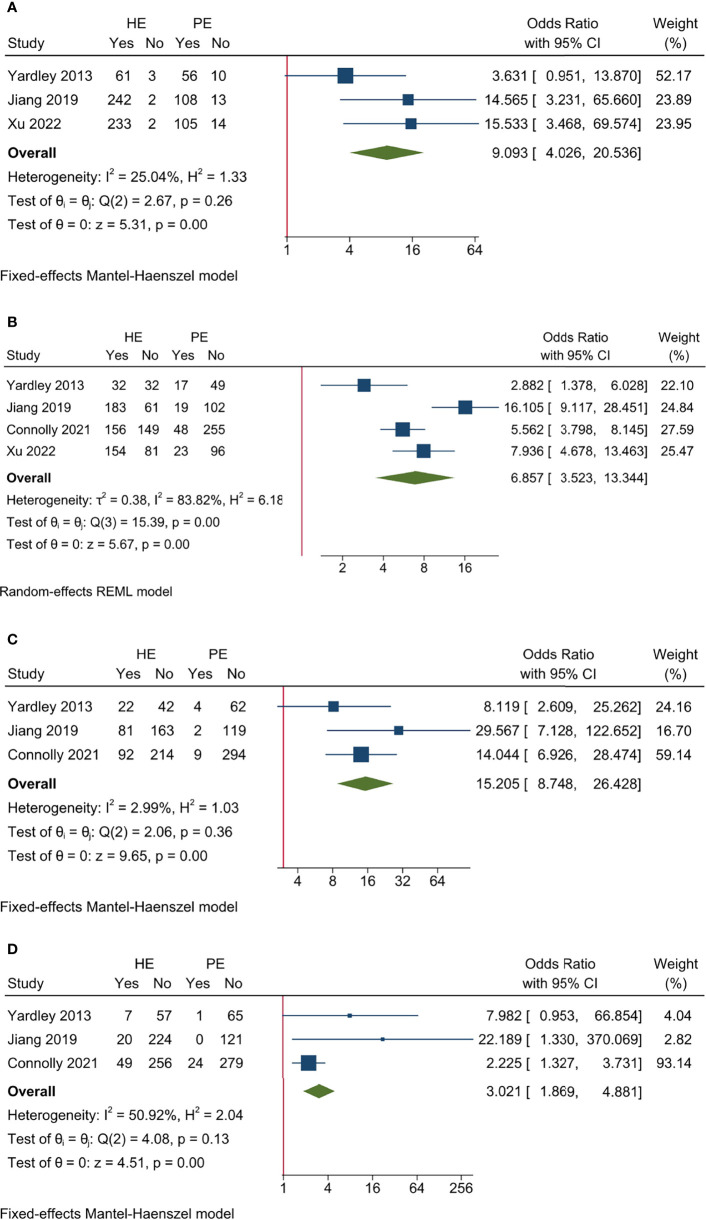
Pooled results for AE endpoints of included studies. **(A)** All AE; **(B)** Grade ≥3 AE; **(C)** dose modification due to AE; **(D)** treatment discontinuation due to AE.

**Figure 4:**
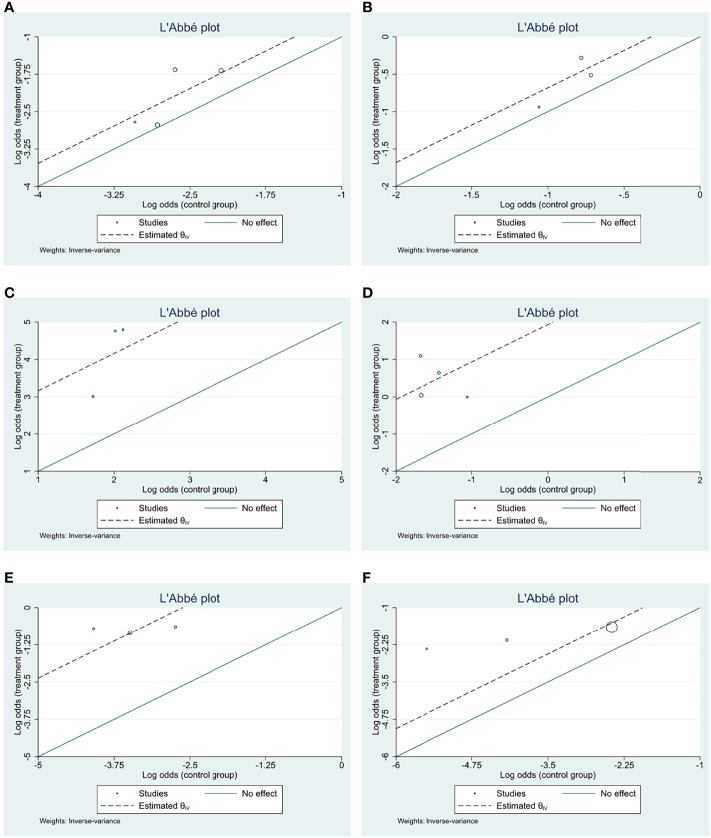
L’Abbé plots for efficacy and AE endpoints of included studies. **(A)** ORR; **(B)** CBR; **(C)** all AE; **(D)** Grade ≥3 AE; **(E)** dose modification due to AE; **(F)** treatment discontinuation due to AE.

Three studies reported AE rate and no significant heterogeneity existed among included studies (*I* ^2^ = 25.04%, Cochrane’s *Q p* = 0.26). The overall AE rates were 98.57% and 87.83% for HE and PE groups, respectively, and the HE regimen had higher AE incidence (OR 9.093, 95% CI = 4.026–20.536, *p* < 0.001) (**Table 3** and **Figure 3A**). The L’Abbé plot is presented in **Figure 4C**.

Three studies had Grade ≥3 AE rate and significant heterogeneity existed among included studies (*I* ^2^ = 83.82%, Cochrane’s *Q p* < 0.001). The overall Grade ≥ 3 AE rates were 61.88% and 17.83% for HE and PE groups, respectively, and the HE regimen had significantly higher Grade ≥ 3 AE (OR 6.857, 95% CI = 3.523–13.344, *p* < 0.001) (**Table 3** and **Figure 3B**). The L’Abbé plot is presented in **Figure 4D**.

Three studies reported DM rate and no significant heterogeneity existed among included studies (*I* ^2^ = 2.99%, Cochrane’s *Q p* = 0.36). The overall DM rate was 31.72% and 3.16% for HE and PE groups, respectively, and the HE regimen was associated with a higher DM rate (OR 15.205, 95% CI = 8.748–26.428, *p* < 0.001) (**Table 3** and **Figure 3C**). The L’Abbé plot is presented in **Figure 4E**.

Three studies reported discontinuation rate and no significant heterogeneity existed among included studies (*I* ^2^ = 50.92%, Cochrane’s *Q p* = 0.13). The overall discontinuation rates were 12.29% and 5.22% for HE and PE groups, respectively, and the HE regimen was associated with higher discontinuation rate (OR 3.021, 95% CI = 1.869–4.881, *p* < 0.001) (**Table 3** and **Figure 3D**). The L’Abbé plot is presented in **Figure 4F**.

### Subgroup Analysis for Entinostat

Entinostat was one of the most widely investigated agents of HDACi, and three out of four included studies focused on entinostat. Hence, we carried out subgroup analysis for entinostat.

**Figure 5:**
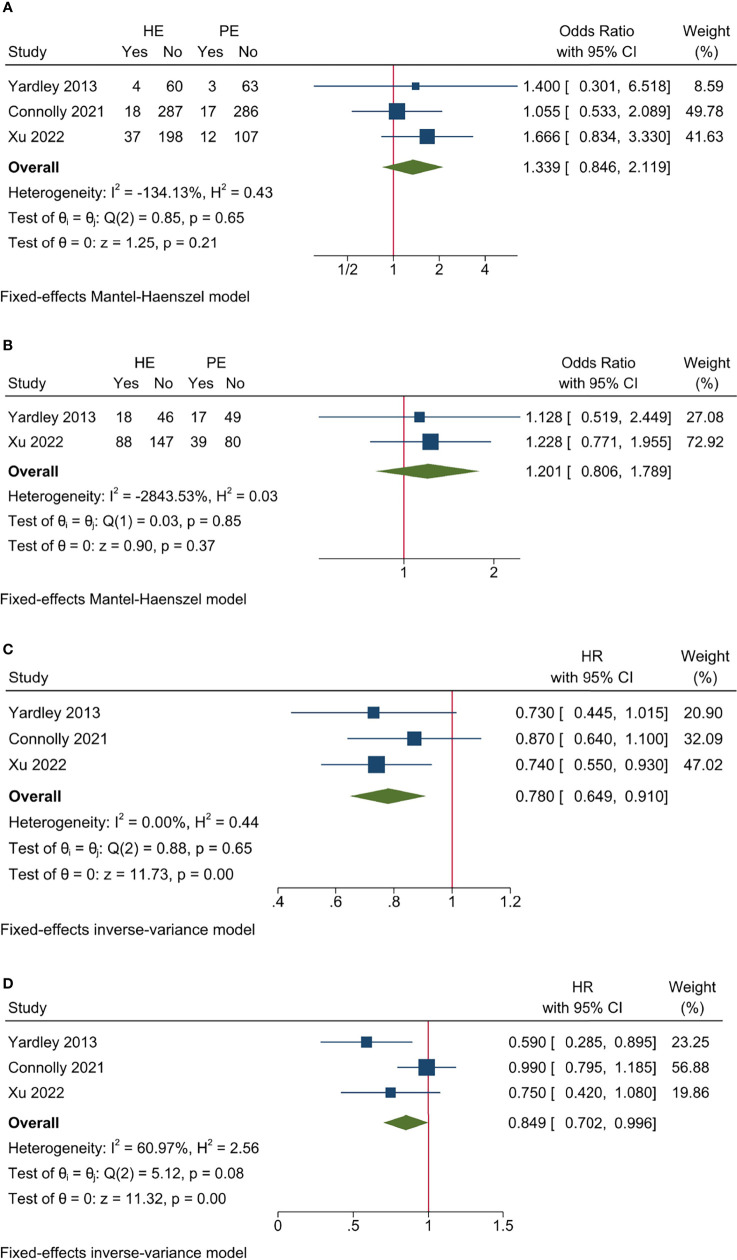
Sensitivity analysis for efficacy endpoints of studies on entinostat. **(A)** ORR; **(B)** CBR; **(C)** PFS; **(D)** OS.

The pooled efficacy data revealed that entinostat had no significant impact on ORR (*I* ^2^ = −134.13%, Cochrane’s *Q p* = 0.65; OR 1.339, 95% CI = 0.846–2.119, *p* = 0.21) and CBR (*I* ^2^ = −2843.53%, Cochrane’s *Q p* = 0.85; OR 1.201, 95% CI = 0.806–1.789, *p* = 0.37) (**Figures 5A, B**). However, entinostat could significantly increase PFS (*I* ^2^ = 0.00%, Cochrane’s *Q p* = 0.65; HR 0.780, 95% CI = 0.649–0.910, *p* < 0.001) and OS (*I* ^2^ = 60.97%, Cochrane’s *Q p* = 0.08; HR 0.849, 95% CI = 0.702–0.996, *p* < 0.001) (**Figures 5C, D**).

**Figure 6:**
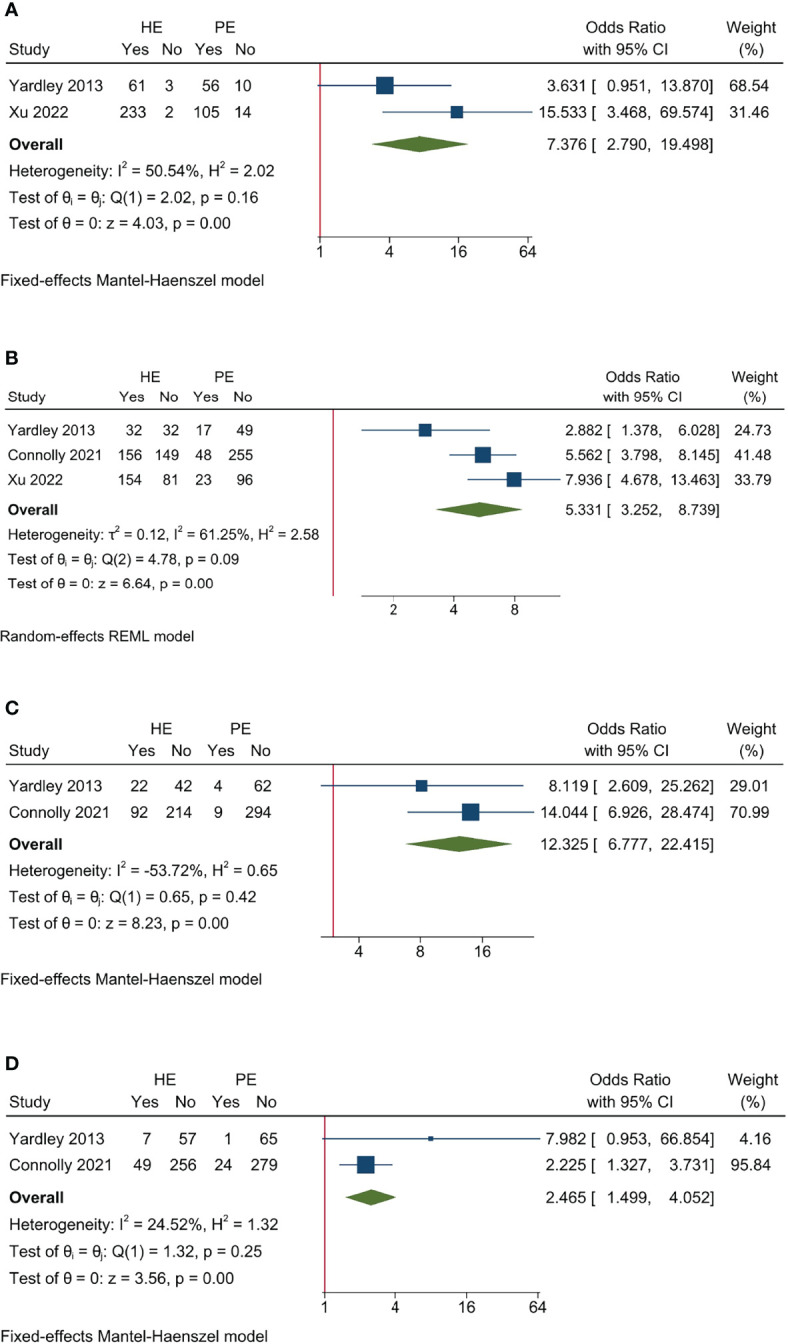
Sensitivity analysis for AE endpoints of studies on entinostat. **(A)** All AE; **(B)** Grade ≥3 AE; **(C)** dose modification due to AE; **(D)** treatment discontinuation due to AE.

The pooled AE data revealed that entinostat had greater toxicity than placebo. It had increasing AE rate (*I* ^2^ = 50.54%, Cochrane’s *Q p* = 0.16; OR 7.736, 95% CI = 2.790–19.498, *p* < 0.001), Grade ≥ 3 AE rate (*I* ^2^ = 61.25%, Cochrane’s *Q p* = 0.09; OR 5.331, 95% CI = 3.252–8.739, *p* < 0.001), DM rate (*I* ^2^ = −53.72%, Cochrane’s *Q p* = 0.42; OR 12.325, 95% CI = 6.777–22.415, *p* < 0.001), and discontinuation rate (*I* ^2^ = 24.52%, Cochrane’s *Q p* = 0.25; HR 2.465, 95% CI = 1.499–4.052, *p* < 0.001) compared to control (**Figure 6**).

### Publication Bias

Potential publication bias was evaluated by Funnel plots with symmetrical appearance (**Supplementary Figure 1**). Egger’s test suggested no significant publication bias for all the endpoints (ORR *p* = 0.94, CBR *p* = 0.57, PFS *p* = 0.90, AE rate *p* = 0.10, Grade ≥3 AE rate *p* = 0.63, DM rate *p* = 0.69, and discontinuation *p* = 0.06) except for OS *p* < 0.05. “Trim-and-fill” analysis for OS showed that observed + imputed studies yielded the same result as observed-only studies with HR 0.849, 95% CI = 0.702–0.996, *p* < 0.001.

## Discussion

ET remains the keystone systemic therapy for advanced HoR+/HER2- breast cancer. Although the emergence of CDKi, mTOR inhibitor, and PI3KCA inhibitor largely prolonged the PFS and OS for HoR+/HER2- MBC patients, acquired resistance remains a significant challenge. HDACi, as a novel therapy that modifies the acetylation on histone and non-histone proteins, has proved its efficacy in hematological malignancies (27), but remains controversial in breast cancer. The present meta-analysis included four randomized controlled studies with 1,457 patients and demonstrated that HDACi had promising efficacy in terms of increasing ORR/CBR and prolonged PFS/OS, but was associated with higher toxicity. Subgroup analysis revealed similar results for entinostat. Entinostat was associated with superior survival (PFS and OS), but higher overall AE rate and Grade ≥ 3 AE rate, and it also caused increasing risk for dose modification and treatment discontinuation.

Our finding was consistent with several previous randomized control trials. A Phase II trial (ENCORE 301) by Yardley et al. proved that the combination of entinostat and ET could improve PFS (4.3 m vs. 2.3 m) and OS (28.1 m vs. 19.8 m) with fatigue and neutropenia as the most frequent Grade 3/4 AE. This combination was associated with increasing risk for treatment discontinuation (11% vs. 2%) (19). A recent ACE trial with another HDACi, tucidinostat, also demonstrated its efficacy in terms of prolonged survival with a similar safety profile (20). Conversely, the E2112 trial showed that the combination of entinostat and exemestane had no significant impact on ORR and survival (21). Given the concern that results of the positive Phase II trial may not necessarily be replicated in the Phase III trial, the Phase III E2112 trial mirrored the design of ENCORE 301 except for enrollment of premenopausal patients and prior fulvestrant/CDKi. The difference in conclusions of ENCORE 301 and E2112 could be attributed to the fact that approximately 30% of the participants received fulvestrant and 30% had prior CDKi. The much heavily pre-treated study population may attenuate the efficacy of HE in E2112. Another possible reason would be the *c-Myc* gene signatures of study population. *c-Myc* was a key impact factor for HDACi sensitivity in various cancers (28, 29). For breast cancer, a study by Tanioka et al. proved that tumor progression was associated with upregulated *c-Myc* gene signatures and *c-Myc* overexpression conferred resistance to entinostat in breast cancer cell lines. *Jun* deletion, which accounted for 17%–23% of luminal breast cancer, usually incurred significantly higher *c-Myc* signature scores with poorer survival. Hence, future trials with *c-Myc* and *Jun* signature as stratification factors would be helpful to refine the appropriate candidate for HDACi.

Generally, combination therapy was associated with increasing toxicity, such as everolimus plus exemestane, which is demonstrated in the BOLERO-2 trial (2). For the safety profile of the HE regimen, the pooled results indicated increasing AE, Grade ≥3 AE, DM, and discontinuation rates compared to control. These results were consistent across all the included individual studies and sensitive analyses with DM rate up to 30%, and more than 10% patients withdrew. Thus, careful patient monitoring and improved physician awareness of HE with relevant AE are warranted. The AE profile was concordant with previously reported data and HDACi class effects (30, 31). It mainly consisted of hematologic toxicities, gastrointestinal disturbances, and fatigue. Electrolyte disturbances were also noted in the HDACi group, which may be attributed to HDACi gastrointestinal toxicity (20, 32).

Heterogeneity investigation focused on the difference between entinostat and tucidinostat. According to the Cochrane *Q* test, all the efficacy and AE endpoints had no significant heterogeneity among included studies (**Figures 2** and **3**). It further strengthened the primary conclusion that HDACi could improve survival in HoR+/HER2- MBC, but was accompanied with enhanced toxicity. Moreover, according to the characteristics of study design, three out of four included studies investigated entinostat, with one study investigating tucidinostat. Tucidinostat and entinostat both belonged to the subtype-selective class of HDACi that had improved risk–benefit profiles compared to non-selective inhibitors (33). Subtype-selective HDACi had an advantage over non-selective HDACi in terms of enhanced immune cell-mediated tumor cell cytotoxicity (34). The difference between tucidinostat and entinostat was an important confounding factor for pooled results. Hence, sensitivity analyses were conducted with studies on entinostat only. Compared to the overall pooled results, the subgroup with entinostat drew similar conclusions in that entinostat benefited PFS and OS with increasing AE, Grade ≥3 AE, DM, and treatment discontinuation rate, but it did not significantly improve ORR and CBR. It implied that tucidinostat may have a stronger effect on reducing tumor burden than entinostat. However, this conclusion should be used with caution and needs further validation due to several other confounding factors in the ACE trial. The ACE trial enrolled generally younger patients (median age: 8 years difference) with less prior ET (34% less) compared with the E2112 trial (21). This less pre-treated population could partially explain the enhanced efficacy of tucidinostat in the ACE trial. Additionally, the ACE trial only recruited Chinese patients, and the ethnic difference between Asian and Caucasian women may also introduce bias to the pooled result. Finally, the study population received no prior CDKi due to drug availability, indicating that it may confer sensitivity to HDACi.

The present meta-analysis had several strengths. First, it was the first meta-analysis with a large study population (four trials with 1,457 patients included) for the HE regimen. It analyzed several efficacy and safety endpoints (ORR, CBR, PFS, OS, AE rate, Grade ≥3 AE, DM rate, and treatment discontinuation) to give a comprehensive overview of the efficacy and toxicity of the HE regimen. Moreover, subgroup analysis provided detailed information on entinostat, which may facilitate clinical decision-making. The present meta-analysis demonstrated that HDACi with ET showed promising efficacy with increasing toxicity, and it may serve as an optional regimen for HoR+/HER2- MBC. The HE regimen had several advantages. First, OS remained the most important endpoint for the assessment of novel agents in advanced cancers, and the OS improvement by HE was a strong indicator for clinical benefit. Secondly, HE may be effective for progressive disease after CDKi, although only limited patients with prior CDKi were included in the study population. Third, HDACi could potentially increase patient compliance, given that HDACi dosing was usually once/twice per week rather than once/twice per day in CDKi. Finally, differences in toxicity profiles between HDACi and other agents may also be crucial for clinical decision-making to deliver personalized treatment. Given that merely 25% of the study population received prior CDKi and fulvestrant, future studies should focus on HE efficacy for CDKi-resistant MBC. Additionally, the novel combination of HDACi and a selective estrogen receptor degrader (such as fulvestrant) still needs further evaluation by a large-scale clinical trial. It was also of great importance to investigate the ethnic difference between Asian and Caucasian patients, and to find effective biomarkers to predict HDACi sensitivity.

Our study had several limitations. First, it was a meta-analysis based on aggregate data rather than individual patient data. The pooled results were subject to publication bias and summary effects should be interpreted carefully with the context of heterogeneity. Second, only one study on tucidinostat was available; there were no subgroup analyses on tucidinostat and other HDACi. Third, given that limited studies were included, meta-regression and subgroup analysis on several critical clinicopathological variables, such as prior fulvestrant and CDKi usage and previous lines of chemotherapy, could not be conducted.

The present meta-analysis demonstrated that the HE regimen improved patient survival in HoR+/HER2- MBC with increasing but manageable toxicity. A large-scale randomized controlled trial would be helpful to further validate the efficacy and safety profile of HDACi and investigate the clinical difference among different HDACi.

## Conclusion

This meta-analysis validated that the HE regimen had superior efficacy over control in terms of improved ORR, CBR, PFS, and OS, but was accompanied with increasing AE. HDACi + ET could serve as an important option for the management of HoR+/HER2- MBC. Future studies may focus on the clinical difference among different HDACi and AE management to enhance tolerability.

## Data Availability Statement

The datasets presented in this study can be found in online repositories. The names of the repository/repositories and accession number(s) can be found in the article/**Supplementary Material**.

## Author Contributions

CW, YZ, QS, and CL designed the project. CW, YL, QS, and CL performed the literature search and data acquisition. CW and YL performed data extraction. FM, HZ, and XH performed the statistical analyses for heterogeneity investigation. CW, HZ, and YZ supported the writing of the paper. All authors contributed to the article and approved the submitted version.

## Funding

This study was funded by Key Projects in the National Science and Technology Pillar Program during the Twelfth Five-year Plan Period (No. 2014BAI08B00), Beijing Municipal Science and Technology Project (No. D161100000816005), State Key Laboratory of Medicinal Chemical Biology (NanKai University) (No. 2019014), and LAM China Non-profit Organization Special Fund for LAM of Zhejiang Women and Children's Foundation (LAM001-202205). The funding agencies had no role in the design or conduct of the study.

## Conflict of Interest

The authors declare that the research was conducted in the absence of any commercial or financial relationships that could be construed as a potential conflict of interest.

## Publisher’s Note

All claims expressed in this article are solely those of the authors and do not necessarily represent those of their affiliated organizations, or those of the publisher, the editors and the reviewers. Any product that may be evaluated in this article, or claim that may be made by its manufacturer, is not guaranteed or endorsed by the publisher.

## Supplementary Material

Supplementary Figure 1 Funnel Plots for publication bias evaluation: **(A)** ORR; **(B)** CBR; **(C)** PFS; **(D)** OS; **(E)** All AE; **(F)** Grade ≥3 AE; **(G)** Dose modification due to AE; **(H)** Treatment discontinuation due to AE.

## References

[1] André F Ciruelos E Rubovszky G Campone M Loibl S Rugo HS . Alpelisib for *PIK3CA* -Mutated, Hormone Receptor–Positive Advanced Breast Cancer. N Engl J Med (2019) 380:1929–40. doi: 10.1056/NEJMoa1813904 31091374

[2] Baselga J Campone M Piccart M Burris HA Rugo HS Sahmoud T . Everolimus in Postmenopausal Hormone-Receptor–Positive Advanced Breast Cancer. N Engl J Med (2012) 366:520–9. doi: 10.1056/NEJMoa1109653 PMC5705195 22149876

[3] Turner NC Slamon DJ Ro J Bondarenko I Im S-A Masuda N . Overall Survival With Palbociclib and Fulvestrant in Advanced Breast Cancer. N Engl J Med (2018) 379:1926–36. doi: 10.1056/NEJMoa1810527 30345905

[4] Sledge GW Toi M Neven P Sohn J Inoue K Pivot X . The Effect of Abemaciclib Plus Fulvestrant on Overall Survival in Hormone Receptor–Positive, ERBB2-Negative Breast Cancer That Progressed on Endocrine Therapy—MONARCH 2: A Randomized Clinical Trial. JAMA Oncol (2020) 6:116. doi: 10.1001/jamaoncol.2019.4782 31563959 PMC6777264

[5] Casimiro MC Velasco-Velázquez M Aguirre-Alvarado C Pestell RG . Overview of Cyclins D1 Function in Cancer and the CDK Inhibitor Landscape: Past and Present. Expert Opin Investig Drugs (2014) 23:295–304. doi: 10.1517/13543784.2014.867017 24387133

[6] Dimitrakopoulos F-I Kottorou A Tzezou A . Endocrine Resistance and Epigenetic Reprogramming in Estrogen Receptor Positive Breast Cancer. Cancer Lett (2021) 517:55–65. doi: 10.1016/j.canlet.2021.05.030 34077785

[7] Jeselsohn R Buchwalter G De Angelis C Brown M Schiff R . ESR1 Mutations—a Mechanism for Acquired Endocrine Resistance in Breast Cancer. Nat Rev Clin Oncol (2015) 12:573–83. doi: 10.1038/nrclinonc.2015.117 PMC4911210 26122181

[8] Picon-Ruiz M Morata-Tarifa C Valle-Goffin JJ Friedman ER Slingerland JM . Obesity and Adverse Breast Cancer Risk and Outcome: Mechanistic Insights and Strategies for Intervention. CA Cancer J Clin (2017) 67:378–97. doi: 10.3322/caac.21405 PMC5591063 28763097

[9] Bhattacharjee D Shenoy S Bairy KL . DNA Methylation and Chromatin Remodeling: The Blueprint of Cancer Epigenetics. Scientifica (2016) 2016:6072357. doi: 10.1155/2016/6072357 27119045 PMC4826949

[10] Ramaiah MJ Tangutur AD Manyam RR . Epigenetic Modulation and Understanding of HDAC Inhibitors in Cancer Therapy. Life Sci (2021) 277:119504. doi: 10.1016/j.lfs.2021.119504 33872660

[11] Liang XH Jackson S Seaman M Brown K Kempkes B Hibshoosh H . Induction of Autophagy and Inhibition of Tumorigenesis by Beclin 1. Nature (1999) 402:672–6. doi: 10.1038/45257 10604474

[12] Newbold A Falkenberg KJ Prince HM Johnstone RW . How do Tumor Cells Respond to HDAC Inhibition? FEBS J (2016) 283:4032–46. doi: 10.1111/febs.13746 27112360

[13] Condorelli F Gnemmi I Vallario A Genazzani AA Canonico PL . Inhibitors of Histone Deacetylase (HDAC) Restore the P53 Pathway in Neuroblastoma Cells. Br J Pharmacol (2008) 153:657–68. doi: 10.1038/sj.bjp.0707608 PMC2259214 18059320

[14] Borbone E Berlingieri MT De Bellis F Nebbioso A Chiappetta G Mai A . Histone Deacetylase Inhibitors Induce Thyroid Cancer-Specific Apoptosis Through Proteasome-Dependent Inhibition of TRAIL Degradation. Oncogene (2010) 29:105–16. doi: 10.1038/onc.2009.306 19802013

[15] Ellis L Hammers H Pili R . Targeting Tumor Angiogenesis With Histone Deacetylase Inhibitors. Cancer Lett (2009) 280:145–53. doi: 10.1016/j.canlet.2008.11.012 PMC2814368 19111391

[16] El-Naggar AM Somasekharan SP Wang Y Cheng H Negri GL Pan M . Class I HDAC Inhibitors Enhance YB-1 Acetylation and Oxidative Stress to Block Sarcoma Metastasis. EMBO Rep (2019) 20:e48375. doi: 10.15252/embr.201948375 31668005 PMC6893361

[17] Garmpi A Garmpis N Damaskos C Valsami S Spartalis E Lavaris A . Histone Deacetylase Inhibitors as a New Anticancer Option: How Far can We Go With Expectations? Delivery Systems. J BUON Off J Balk Union Oncol (2018) 23:846–61. 30358185

[18] Munster PN Thurn KT Thomas S Raha P Lacevic M Miller A . A Phase II Study of the Histone Deacetylase Inhibitor Vorinostat Combined With Tamoxifen for the Treatment of Patients With Hormone Therapy-Resistant Breast Cancer. Br J Cancer (2011) 104:1828–35. doi: 10.1038/bjc.2011.156 PMC3111195 21559012

[19] Yardley DA Ismail-Khan RR Melichar B Lichinitser M Munster PN Klein PM . Randomized Phase II, Double-Blind, Placebo-Controlled Study of Exemestane With or Without Entinostat in Postmenopausal Women With Locally Recurrent or Metastatic Estrogen Receptor-Positive Breast Cancer Progressing on Treatment With a Nonsteroidal Aromatase Inhibitor. J Clin Oncol (2013) 31:2128–35. doi: 10.1200/JCO.2012.43.7251 PMC4881332 23650416

[20] Jiang Z Li W Hu X Zhang Q Sun T Cui S . Tucidinostat Plus Exemestane for Postmenopausal Patients With Advanced, Hormone Receptor-Positive Breast Cancer (ACE): A Randomised, Double-Blind, Placebo-Controlled, Phase 3 Trial. Lancet Oncol (2019) 20:806–15. doi: 10.1016/S1470-2045(19)30164-0 31036468

[21] Connolly RM Zhao F Miller KD Lee M-J Piekarz RL Smith KL . E2112: Randomized Phase III Trial of Endocrine Therapy Plus Entinostat or Placebo in Hormone Receptor–Positive Advanced Breast Cancer. A Trial of the ECOG-ACRIN Cancer Research Group. J Clin Oncol (2021) 39:3171–81. doi: 10.1200/JCO.21.00944 PMC8478386 34357781

[22] STROBE Statement . Available at: https://www.strobe-statement.org/index.php?id=available-checklists (Accessed August 8, 2020).

[23] von Elm E Altman DG Egger M Pocock SJ Gøtzsche PC Vandenbroucke JP . The Strengthening the Reporting of Observational Studies in Epidemiology (STROBE) Statement: Guidelines for Reporting Observational Studies. Lancet Lond Engl (2007) 370:1453–7. doi: 10.1016/S0140-6736(07)61602-X 18064739

[24] Wang C-J Xu Y Lin Y Zhu H-J Zhou Y-D Mao F . Platinum-Based Neoadjuvant Chemotherapy for Breast Cancer With BRCA Mutations: A Meta-Analysis. Front Oncol (2020) 10:592998. doi: 10.3389/fonc.2020.592998 33304851 PMC7693629

[25] Tierney JF Stewart LA Ghersi D Burdett S Sydes MR . Practical Methods for Incorporating Summary Time-to-Event Data Into Meta-Analysis. Trials (2007) 8:16. doi: 10.1186/1745-6215-8-16 17555582 PMC1920534

[26] Xu B Zhang Q Hu X Li Q Sun T Li W . Abstract GS1-06: A Randomized Control Phase III Trial of Entinostat, a Once Weekly, Class I Selective Histone Deacetylase Inhibitor, in Combination With Exemestane in Patients With Hormone Receptor Positive Advanced Breast Cancer. Cancer Res (2022) 82:GS1–06. doi: 10.1158/1538-7445.SABCS21-GS1-06

[27] Jones PA Issa J-PJ Baylin S . Targeting the Cancer Epigenome for Therapy. Nat Rev Genet (2016) 17:630–41. doi: 10.1038/nrg.2016.93 27629931

[28] Pei Y Liu K-W Wang J Garancher A Tao R Esparza LA . HDAC and PI3K Antagonists Cooperate to Inhibit Growth of MYC-Driven Medulloblastoma. Cancer Cell (2016) 29:311–23. doi: 10.1016/j.ccell.2016.02.011 PMC4794752 26977882

[29] Nebbioso A Carafa V Conte M Tambaro FP Abbondanza C Martens J . C-Myc Modulation and Acetylation Is a Key HDAC Inhibitor Target in Cancer. Clin Cancer Res Off J Am Assoc Cancer Res (2017) 23:2542–55. doi: 10.1158/1078-0432.CCR-15-2388 27358484

[30] Whittaker SJ Demierre M-F Kim EJ Rook AH Lerner A Duvic M . Final Results From a Multicenter, International, Pivotal Study of Romidepsin in Refractory Cutaneous T-Cell Lymphoma. J Clin Oncol Off J Am Soc Clin Oncol (2010) 28:4485–91. doi: 10.1200/JCO.2010.28.9066 20697094

[31] Tan J Cang S Ma Y Petrillo RL Liu D . Novel Histone Deacetylase Inhibitors in Clinical Trials as Anti-Cancer Agents. J Hematol Oncol J Hematol Oncol (2010) 3:5. doi: 10.1186/1756-8722-3-5 20132536 PMC2827364

[32] Rasheed W Bishton M Johnstone RW Prince HM . Histone Deacetylase Inhibitors in Lymphoma and Solid Malignancies. Expert Rev Anticancer Ther (2008) 8:413–32. doi: 10.1586/14737140.8.3.413 18366289

[33] Yang F Zhao N Ge D Chen Y . Next-Generation of Selective Histone Deacetylase Inhibitors. RSC Adv (2019) 9:19571–83. doi: 10.1039/C9RA02985K PMC9065321 35519364

[34] Ning Z-Q Li Z-B Newman MJ Shan S Wang X-H Pan D-S . Chidamide (CS055/HBI-8000): A New Histone Deacetylase Inhibitor of the Benzamide Class With Antitumor Activity and the Ability to Enhance Immune Cell-Mediated Tumor Cell Cytotoxicity. Cancer Chemother Pharmacol (2012) 69:901–9. doi: 10.1007/s00280-011-1766-x 22080169

