# Supplementary figures and images for: Efficacy and Safety Profile of Histone Deacetylase Inhibitors for Metastatic Breast Cancer: A Meta-Analysis
Source: Front Oncol. 2022 May 31;12:901152. doi: 10.3389/fonc.2022.901152 (PMC9192957; doi:10.3389/fonc.2022.901152)

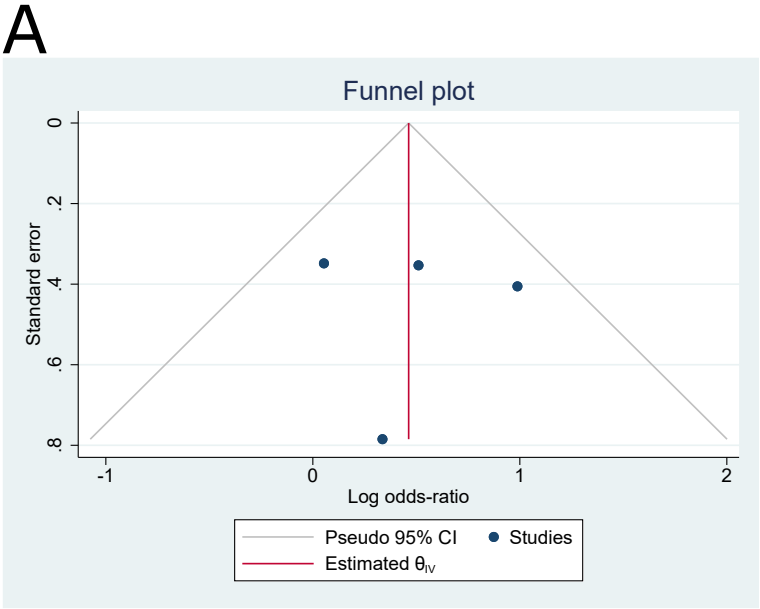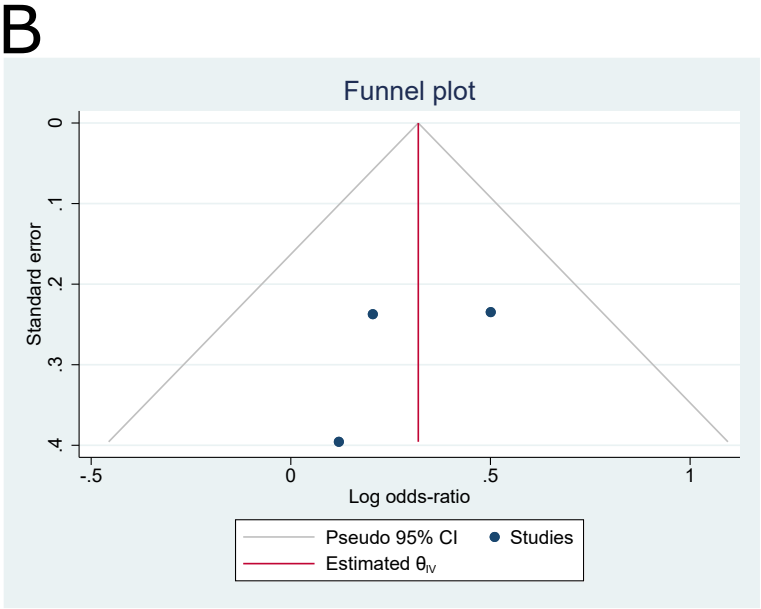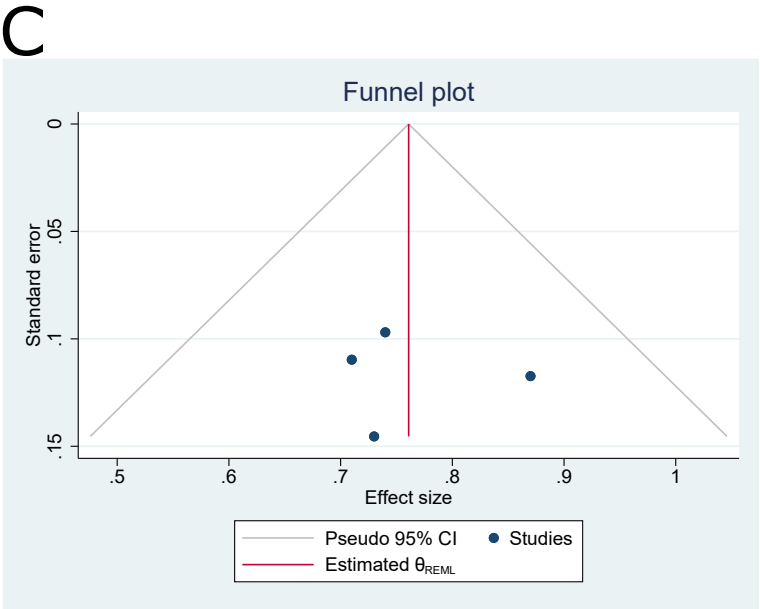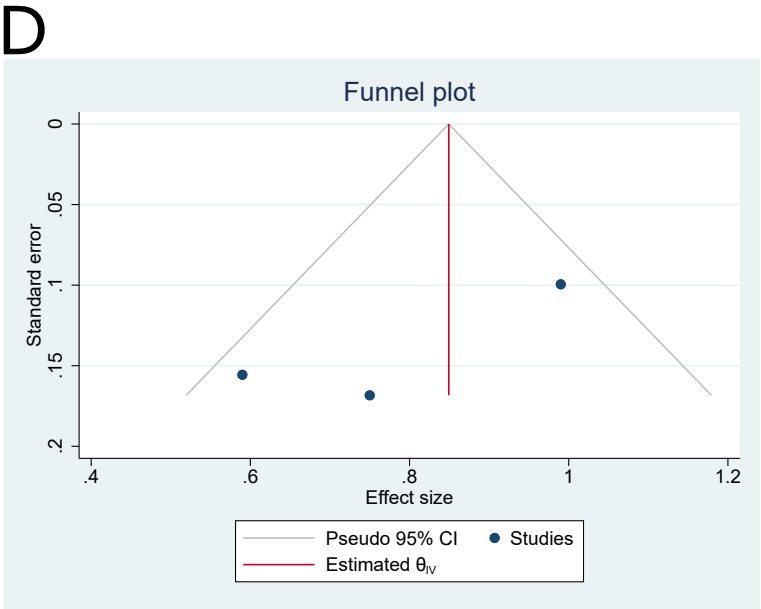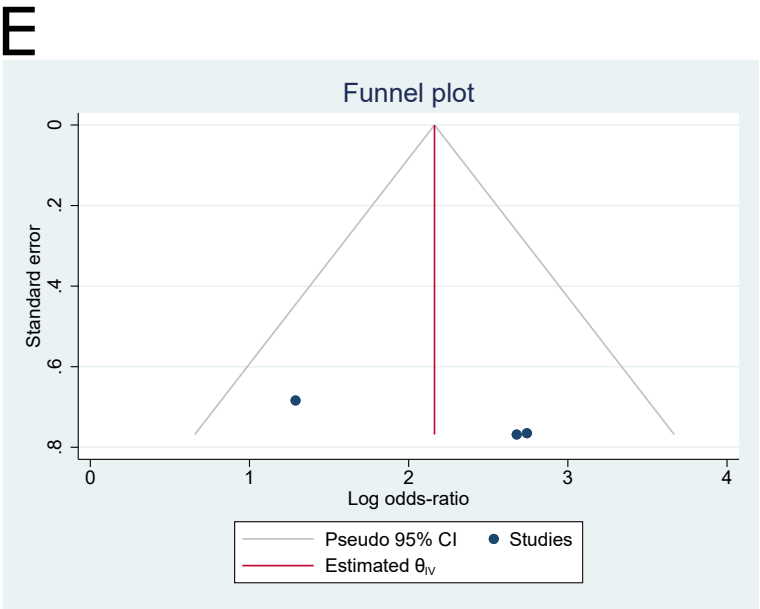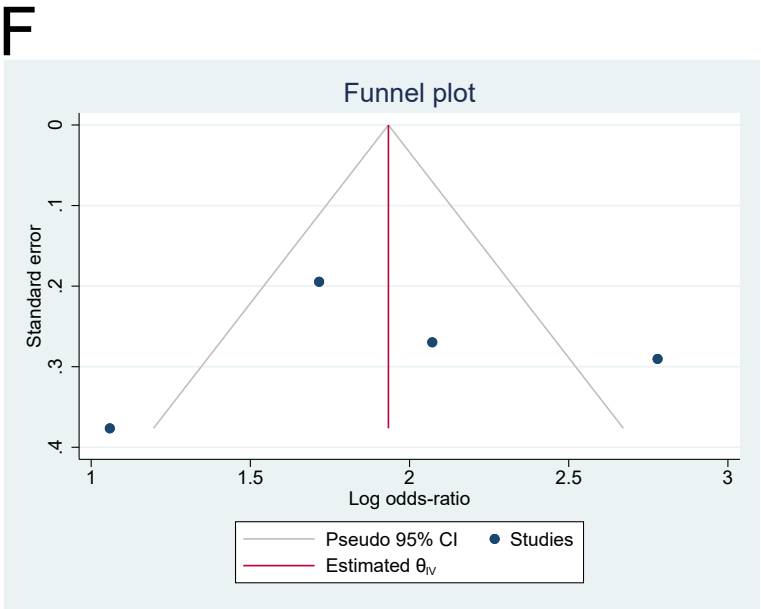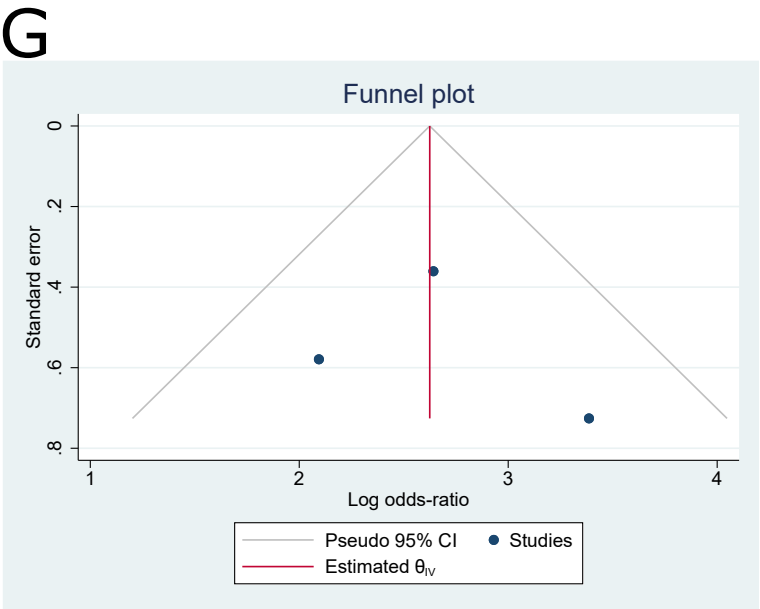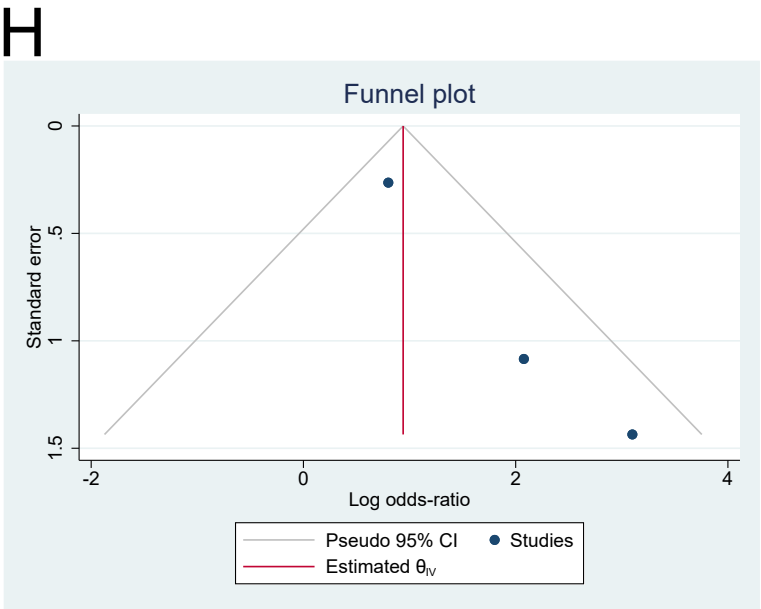

Supplement: Supplementary Figure 1 — Funnel Plots for publication bias evaluation: (A) ORR; (B) CBR; (C) PFS; (D) OS; (E) All AE; (F) Grade ≥3 AE; (G) Dose modification due to AE; (H) Treatment discontinuation due to AE. [file DataSheet_1.pdf]
