# Supplementary material for: Efficacy and Safety Profile of Histone Deacetylase Inhibitors for Metastatic Breast Cancer: A Meta-Analysis
Source: Front Oncol. 2022 May 31;12:901152. doi: 10.3389/fonc.2022.901152 (PMC9192957; doi:10.3389/fonc.2022.901152)
Supplement: Supplementary file 2 [file Table_1.docx]

**Supplement Table 1 Quality score for included studies**

| **Study** | **WCJ** | **LY** | **Average score** |
| --- | --- | --- | --- |
| **Yardley 2013**  **(ENCORE 301)** | 94.55 | 96.36 | 95.46 |
| **Jiang 2019**  **(ACE)** | 95.45 | 93.64 | 94.55 |
| **Connolly 2021**  **(E2112)** | 95.91 | 96.82 | 96.37 |
| **Xu 2022** | 96.36 | 97.27 | 96.82 |
